# Hitting the Sweet Spot: Glycans as Targets of Fungal Defense Effector Proteins

**DOI:** 10.3390/molecules20058144

**Published:** 2015-05-06

**Authors:** Markus Künzler

**Affiliations:** Institute of Microbiology, Department of Biology, ETH Zürich, Vladimir-Prelog-Weg 4, 8093 Zürich, Switzerland; E-Mail: mkuenzle@ethz.ch; Tel.: +41-44-632-4925; Fax: +41-44-632-1148

**Keywords:** antibiosis, resistance, fungi, nematodes, bacteria, lectin, defensin, toxin, glycolipid, glycoprotein

## Abstract

Organisms which rely solely on innate defense systems must combat a large number of antagonists with a comparatively low number of defense effector molecules. As one solution of this problem, these organisms have evolved effector molecules targeting epitopes that are conserved between different antagonists of a specific taxon or, if possible, even of different taxa. In order to restrict the activity of the defense effector molecules to physiologically relevant taxa, these target epitopes should, on the other hand, be taxon-specific and easily accessible. Glycans fulfill all these requirements and are therefore a preferred target of defense effector molecules, in particular defense proteins. Here, we review this defense strategy using the example of the defense system of multicellular (filamentous) fungi against microbial competitors and animal predators.

## 1. Introduction and Scope of This Review

Filamentous fungi are among the simplest eukaryotic organisms with true multicellularity and tissue differentiation [1]. They lack a vascular system and thus nutrients are transported within the organism exclusively via direct cell-cell contacts. As a matter of fact, the cytoplasm of the cellular compartments of a filamentous fungus forms a continuum due to pores in the separating cell walls. In some fungal phyla (Zygomycota), separating cell walls are even lacking. Fungi are heterotrophic organisms that feed by secretion of hydrolytic enzymes and absorption of the hydrolysis products through cell wall and plasma membrane. In order to maximize the surface for absorption, multicellular fungi arrange their cells in linear arrays, referred to as hyphae or filaments, that are able to branch and fuse, and penetrate the substrate as a loose, three-dimensional network, referred to as mycelium. In addition to this long-lived and constantly renewed vegetative mycelium, hyphae of dikaryotic fungi can form compact, short-lived and spore-producing structures, referred to as fruiting bodies, during their sexual reproduction cycle. The group of dikaryotic fungi comprises the phyla Ascomycota and Basidiomycota and covers over 98% of all known fungal species. In addition to the sexual reproduction by spore production in fruiting bodies, many of these fungi, in particular Ascomycota, also reproduce by producing mitotic spores on the vegetative mycelium.

Depending on their ecological niche and their preferred substrate, fungi are exposed to a variety of antagonists. Due to its high surface to volume ratio, the vegetative mycelium of a fungus is very susceptible to other microorganisms that compete for the same nutrients and may even feed on the degradation products released by the action of the hydrolytic enzymes secreted by the fungus. Very nutrient-rich substrates, such as the dung of herbivores, are usually colonized by a plethora of competing microorganisms [2]. The lack of motility and the high nutrient content, on the other hand, makes both the fungal vegetative mycelium and the fruiting bodies very attractive food sources for animal predators. Accordingly, soil inhabiting fungi have been shown to be an important food source for soil arthropods and nematodes [3].

**Figure 1:**
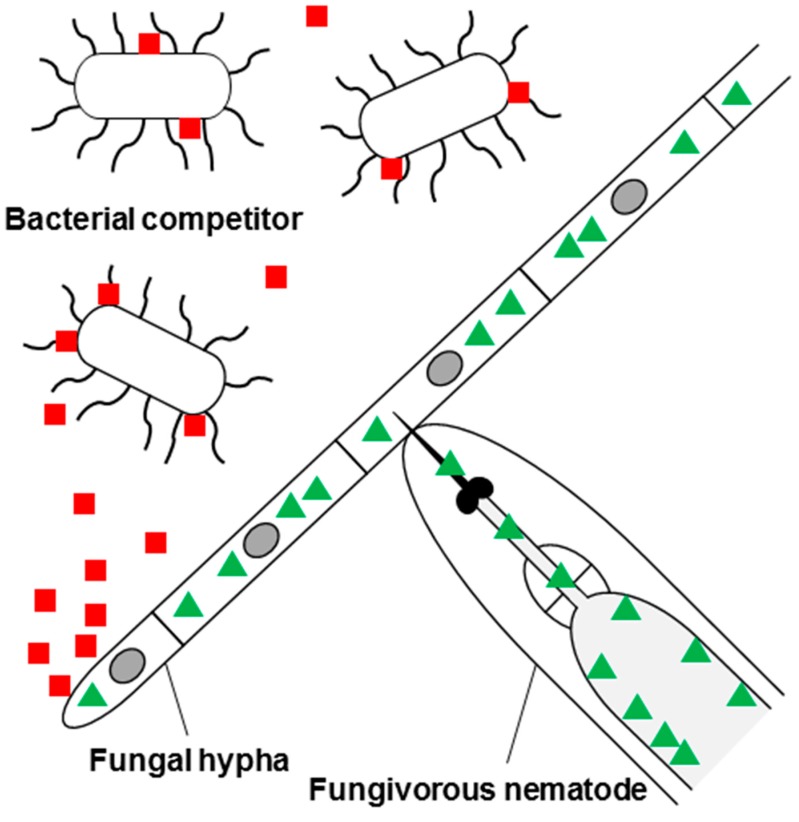
Hypothesis on the localization of glycan-binding fungal defense effector proteins in relation to the type of antagonist. Fungal effector proteins against bacterial competitors (red squares) are secreted and bind to surface glycoepitopes of the bacterial cells, whereas effector proteins against predatory nematodes (green triangles) are kept in the cytoplasm of the fungal cells, ingested by the nematode upon feeding and bind to surface glycoepitopes of intestinal epithelial cells of the nematode. See text for details.

Fungi have evolved different defense strategies to compete with other microorganisms for nutrients and protect themselves from predation by animals. Similar to plants, the main defense strategy of fungi is chemical defense *i.e.*, the production of molecules impairing the development, growth or viability of the antagonists, by the fungus [4]. These defense effector molecules include small molecules (secondary metabolites) [5], peptides (ribosomally or non-ribosomally synthesized) [6,7,8] and proteins [9], and act by binding to specific target molecules in the antagonists. The binding of the effector to the target molecule either inhibits or triggers cellular processes ultimately leading to impairment of growth and development and/or viability of the antagonist. We hypothesize that effector molecules against microbial competitors are generally secreted whereas molecules against animal predators are usually stored within the fungal cells and only released upon predation (Figure 1). According to this hypothesis, antimicrobial effectors are supposed to find their targets on the surface of the microbial cells including the cell wall and the outer surface of the plasma membrane, whereas effectors against animal predators bind to targets in the digestive tract of the animal. The most famous examples of fungal defense effectors supporting this hypothesis are the β-lactam antibiotic penicillin produced by some *Penicillium* species [10] and the cytotoxic, ribosomally synthesized bicyclic octapeptide α-amanitin produced by some *Amanita*, *Galerina*, *Conocybe* and *Lepiota* species [11]. Penicillin is secreted and binds and inhibits extracellular enzymes involved in peptidoglycan biosynthesis, an essential and conserved process in all bacteria [12,13]. On the contrary, α-amanitin is only released from the fungal cell upon predation and enters epithelial cells of the digestive tract of metazoan predators where it binds and inactivates the essential and conserved nuclear enzyme RNA polymerase II of metazoans [14]. The RNA polymerase II of the mushroom is insensitive to this toxin [15].

In contrast to the adaptive immune system of vertebrates, which is able to produce a theoretically unlimited number of effector molecules (antibodies) [16], a defense strategy relying solely on innate defense effectors has the intrinsic problem that the repertoire of effector molecules that can be encoded in the genome is limited whereas the potential diversity of antagonists is huge. In order to alleviate this problem, organisms relying solely on innate defense have evolved effector molecules that are directed against target epitopes displayed by and therefore effective against different antagonists. Accordingly, some effector molecules, like penicillin and α-amanitin, target highly conserved protein epitopes that can be hardly changed by the antagonist without losing essential functions. Most defense effector molecules—in particular proteins—, however, target specific glyco- rather than protein epitopes [17]. These glycoepitopes are often conserved between different antagonists of a specific taxon and sometimes even across taxa [18,19,20]. In contrast to the protein epitopes targeted by defense effectors, these glycoepitopes are usually not essential for the viability of these organisms. In this review, we give an overview of the characterized fungal defense proteins that have been shown to target glycoepitopes and highlight some general features of this defense strategy. Glycan-targeting killer toxins, produced by some yeast strains, were omitted from this review because they are encoded by viruses [21] and therefore not regarded as intrinsic part of the fungal defense system.

## 2. Fungal Defense Effector Proteins Targeting Fungal Glycoepitopes

There are not many examples of characterized fungal defense proteins targeting glycoepitopes of other fungi (Table 1). According to above hypothesis, these antifungal effector proteins would be secreted by the defending fungus and be directed glycoepitopes on the surface of the target fungus. These glycoepitopes must be part of the various polysaccharides or glycoproteins that constitute the fungal cell wall or glycolipids on the fungal cell membrane. The conservation of most of these glycoconjugates between different fungal species and phyla [22] makes it difficult for a fungus to evolve effector proteins that target competing fungi and leave its own cells unaffected.

### 2.1. Defensins

One rather well characterized set of fungal defense proteins with antifungal activity is a family of highly basic and cysteine-stabilized defensin-like proteins, AntiFungal Protein (AFP) from *Aspergillus giganteus* [23], Penicillium Antifungal Protein (PAF) from *Penicillium chrysogenum* [24], Bubble Protein (BP) from *Penicillium brevicompactum* Dierckx and their homologs in other ascomycetes [25]. Defensins form the largest family of antimicrobial peptides (AMPs) and are characterized by a net positive charge, antiparallel β-sheets and/or α-helices stabilized by multiple disulfide bridges [26,27]. The defensin family includes proteins with antibacterial (see Section 3.1), antifungal and cytotoxic activity from all domains of life. AFP, PAF and BP form a separate class of defensin-like proteins as their structure differs from the structure of classical defensin-like proteins mentioned below. Whereas AFP and their homologs consist of five antiparallel β-sheets forming a β-barrel, the core of classical defensin-like proteins is built by two antiparallel β-sheets forming a so-called γ-loop [28]. Among these non-classical antifungal defensin-like proteins, AFP is best characterized in terms of target specificity and toxicity mechanism. The protein has been demonstrated to bind to the polysaccharide chitin *in vitro* and to specifically inhibit the activity of chitin synthase III and V [23]. Latter activity appears to determine the specificity of the protein since filamentous fungi, which have these enzyme classes, are AFP-sensitive, whereas yeasts, which do not have chitin synthase III and V, are insensitive. The exact mechanism of the inhibition of these chitin synthases by AFP, however, is not clear since AFP is supposed to act extracellularly and the actual biosynthesis of chitin by chitin synthase takes place on the cytoplasmic side of the plasma membrane. Besides chitin and its biosynthetic machinery, glucosylceramide in the plasma membrane of the target fungus may act as second target of AFP as reduced levels of this fungal-specific glycosphingolipid resulted in reduced AFP-susceptibility of the fungus [29]. Fungal-specific glycosphingolipids, including glucosylceramide and mannosylinositol-phosphorylceramide (MIPC), have been shown to be targeted by antifungal defensins from plants and animals [30]. In addition to their suggested role in antifungal defense, these defensin-like proteins may also play a role in asexual development [31], a role that has been proposed for some of the classical defensin-like proteins from fungi [32].

### 2.2. LysM Effector Proteins

Another group of potential antifungal defense proteins from fungi are LysM-domain-containing proteins [33]. LysM-domains are found in proteins from all kingdoms of life, are approximately 50 amino acids long, have a βααβ-structure and were shown to bind N-acetylglucosamine-containing glycans, such as chitin, chitin-like compounds and peptidoglycan [34]. The domains can be found as parts of cell wall-modifying enyzmes as well as of proteins without additional domains. In fungi, LysM-domains can be found either in combination with a fungal-specific (subgroup C) chitinase domain or in secreted proteins that consist solely of single or multiple LysM-domains. These proteins are referred to as LysM effector proteins and have been shown to dampen the plant defense against plant pathogenic fungi by masking fungal chitooligosaccharides from plant defense receptors and chitinases [35]. A recent sequence comparison of available LysM-domain sequences from fungi showed that there is, besides the characterized family, a separate family of cysteine-stabilized LysM effector proteins that may have different carbohydrate-specificities and functions [33]. Based on the known specificity of LysM-domains for chitin and peptidoglycan, a possible function of these proteins is the defense of fungi against fungal and bacterial competitors. At the moment, there is, however, no experimental evidence for such a function.

### 2.3. Thaumatin-Like Proteins

Thaumatin-like proteins (TLPs) form a large and divergent protein family with members in plants, animals and fungi [36]. The proteins share a sequence of approximately 200 amino acids including a highly conserved family signature that is similar to thaumatin, a sweet-tasting protein isolated from the fruit of the West African rain forest shrub *Thaumatococcus daniellii*. According to the available structures of several plant representatives of this protein family, the polypeptide is folded into three domains, the largest forming a lectin-like β-barrel. The thaumatin fold is stabilized, similar to defensins and some fungal LysM-proteins, by eight disulfide bridges. Despite the structural information, the function of thaumatin-like proteins is unclear and may be diverse. In plants, the protein family is also known as pathogenesis-related protein family 5 (PR5) due to its inducibility upon various types of stress including attack by plant pathogens [37]. Accordingly, several TLPs from plants and fungi have been shown to have antifungal activity and to bind or even hydrolyze β-1,3-glucans, a common polysaccharide of the fungal cell wall [38,39]. At the moment, it is not clear whether the binding or hydrolysis of β-1,3-glucans by TLPs is related to their antifungal activity and whether the fungal representatives of this protein family have a role in defense against fungal competitors or, as recently suggested, an endogenous role in fruiting body senescence [39,40].

### 2.4. Lectins

Several mannose-binding lectins from plants and animals have been shown to inhibit the growth of fungi by binding to mannans attached to glycoproteins in the fungal cell wall [41,42,43]. In contrast, there is, to our knowledge, only a single report about an antifungal lectin, besides above mentioned LysM effector proteins, isolated from a fungus. Amano and coworkers reported that the fucose-binding lectin AAL from *Aleuria aurantia*, which was also shown to have insecticidal and nematotoxic activity (see paragraph 4.1), exhibited antifungal activity against the zygomycete *Mucor racemosus* [44]. The mechanism of the lectin-mediated growth inhibition of fungi is not clear. As one possibility, lectin-binding might interfere with the remodeling of the various polysaccharides in the preexisting fungal cell wall during spore germination or hyphal branching [45,46]—a model that might also apply to LysM effector proteins.

**Table 1:** List of characterized and hypothetical glycan-binding fungal defense proteins described in this review. See text for details.

Protein	Type	Producing Fungus	Target Organism		Target Polysaccharide/Glycoconjugate	Target Glycoepitope (*in vitro*/in vivo)	References
AFP	Non-classical defensin-like	*Aspergillus giganteus*	Filamentous fungi		Chitin Glucosylceramide	(GlcNAc-β1,4)_n_-GlcNAc Glc	[23]
PAF	Non-classical defensin-like	*Penicillium chrysogenum*	Filamentous fungi		Chitin? Glucosylceramide?	*(GlcNAc-β1,4)_n_-GlcNAc?* *Glc?*	[24]
BP	Non-classical defensin-like	*Penicillium brevicompactum*	Filamentous fungi		Chitin? Glucosylceramide?	*(GlcNAc-β1,4)_n_-GlcNAc?* *Glc?*	[25]
	LysM-effector	Various	Filamentous fungi Gram-positive bacteria?		Chitin Peptidoglycan?	*(GlcNAc-β1,4)_n_-GlcNAc* *(MurNAc-β1,4-GlcNAc)_n_?*	[33]
	Thaumatin-like	Various	Filamentous fungi		β1,3-glucans	*(Glc-β1,3)_n_-Glc*	[39]
AAL	Hololectin	*Aleuria aurantia*	Zygomycetes Insects Nematodes Amoeba		Fucose-containing polysaccharides Fucose-containing N- and/or O-glycans?	Fuc-α1,x-X	[44,47,48,49]
Plectasin	Csαβ defensin-like	*Pseudoplectania nigrella*	Gram-positive bacteria		Lipid II	?	[6]
Eurocin	Csαβ defensin-like	*Eurotium amstelodami*	Gram-positive bacteria		Lipid II	?	[50]
Micasin	Csαβ defensin-like	*Microsporum canis*	Gram-positive and -negative bacteria		Lipid II	?	[51]
Copsin	Csαβ defensin-like	*Coprinopsis cinerea*	Gram-positive bacteria		Lipid II	?	[52]
	GH24-lysozyme	Various	Gram-positive bacteria		Peptidoglycan	*(MurNAc-β1,4-GlcNAc)_n_*	
	GH25-lysozyme	*Chalaropsis* sp.	Gram-positive bacteria		Peptidoglycan	*(MurNAc-β1,4-GlcNAc)_n_*	[53]
	Ceratoplatanin	Various	Filamentous fungi? Gram-positive bacteria?		Chitin? Peptidoglycan?	*(GlcNAc-β1,4)_n_-GlcNAc?* *(MurNAc-β1,4-GlcNAc)_n_?*	[54,55]
XCL	Hololectin	*Xerocomus chrysenteron*	Insects Nematodes		N- and/or O-glycans	*Gal-β1,3-GalNAc* and GlcNAc-β1,2-Man	[47,56,57]
TAP1	Hololectin	*Sordaria macrospora*	Insects Nematodes Amoeba	O-glycans?		*Gal-β1,3-GalNAc*	[47,57]
CCL2	Hololectin	*Coprinopsis cinerea*	Insects Nematodes	N-glycan core		GlcNAc-β1,4(Fuc-α1,3)-GlcNAc	[19,57]
CNL	Hololectin	*Clitocybe nebularis*	Mammalian cells Nematodes Amoeba	O-glycans?		*GalNAc*	[47,58]
MPL	Hololectin	*Macrolepiota procera*	Nematodes	N- and/or O-glycans?		*Gal-β1,4-GlcNAc*	[59]
SSA	Hololectin	*Sclerotinia sclerotiorum*	Insects Amoeba	N- and/or O-glycans?		*GalNAc/Gal*	[47,60]
RSA	Hololectin	*Rhizoctonia solani*	Insects	N- and/or O-glycans?		*GalNAc/Gal*	[61,62,63]
CGL1/2	Hololectin	*Coprinopsis cinerea*	Insects Nematodes Amoeba	N- and/or O-glycans? N-glycan core N- and/or O-glycans?		*Gal-β1,4-Glc* *Gal-β1,4-GlcNAc* Gal-β1,4-Fuc	[47,57]
Tectonin2	Hololectin	*Laccaria bicolor*	Nematodes	N-glycan antenna		2-O-Me-Fuc/3-O-Me-Man	[20]
MOA	Chimerolectin	*Marasmius oreades*	Nematodes	Glycosphingolipids		Gal-α1,3-GalNAc	[64,65,66]
LSL	Chimerolectin	*Laetiporus sulphureus*	Mammalian cells	?		*Gal-β1,4-Glc* *Gal-β1,4-GlcNAc*	[67,68]

## 3. Fungal Defense Effector Proteins Targeting Bacterial Glycoepitopes

The architecture and composition of bacterial and fungal cell walls differ significantly [46,69] which makes it easier for the fungus to evolve proteins that specifically target bacterial cell walls. As one of the main differences, the basic building block of bacterial cell walls, peptidoglycan (murein), is a mixed polymer composed of a polysaccharide chain consisting of β1,4-linked N-acetylglucosamine (GlcNAc) and N-acetylmuramic acid (MurNAc) crosslinked with peptide bridges. In contrast, the fungal cell wall mainly consists of the two homopolymers β1,3-glucan and chitin (β1,4-polymerized GlcNAc). On top of the highly conserved peptidoglycan which is located on the inner side of the bacterial cell wall, there are highly repetitive and variable polysaccharides, lipopolysaccharides (LPS) in case of Gram-negative bacteria and lipoteichoic acids (LTA) in case of Gram-positive bacteria, constituting the outer surface of bacteria. The few characterized fungal defense effector proteins with antibacterial activity mainly address the conserved part of the bacterial cell wall (Table 1). 

### 3.1. Defensins

**Figure 2:**
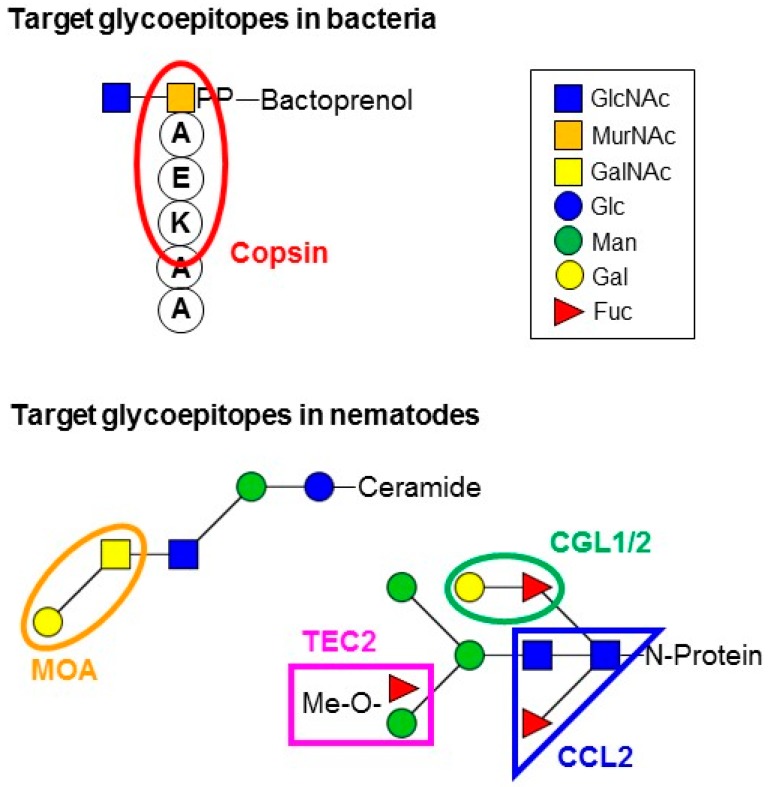
Examples of experimentally verified glycan targets of fungal defense proteins in bacteria and nematodes. The antibacterial Csαβ defensin-like protein copsin from *C. cinerea* binds to the peptide (and eventually also the MurNAc) portion of lipid II [52]. The nematotoxic hololectins CGL1/2 and CCL2 from *C. cinerea* bind to specific modifications of nematode N-glycan cores [18,19], whereas nematotoxic hololectin Tectonin2 (Tec2) from *Laccaria bicolor* binds to 2-O-Me-fucose and/or 3-O-Me-mannose residues on nematode N-glycan antenna (the exact structure of the recognized glycoepitope is not known) [20]. The chimerolectin MOA from *Marasmius oreades* binds to Gal-α1,3-GalNAc epitopes on nematode glycosphingolipids [64]. See text for details.

Analogous to above mentioned, non-classical defensin-like proteins from fungi against fungal competitors, various classical defensin-like proteins with antibacterial activity from both filamentous ascomycota and basidiomycota have been isolated and characterized. These proteins include plectasin from *Pseudoplectania nigrella* [6], eurocin from *Eurotium amstelodami* [50], micasin from *Microsporum canis* [51] and copsin from *Coprinopsis cinerea* [52], respectively. All of these proteins form a cysteine-stabilized fold of two antiparallel β-sheets accompanied by an α-helix (referred to as Csαβ) which reminds of the structure of invertebrate-type defensins [70]. All the characterized examples of these protein family target lipid II, a highly conserved intermediate of peptidoglycan biosynthesis in all bacteria, with slightly different specificities [52,71] (Figure 2). This glycolipid is one of the most favored targets of antibiotics due to its conservation and accessibility on the cell surface of all bacteria [72]. Due to the lack of an outer membrane, Gram-positive bacteria are usually more susceptible to these proteins than Gram-negative bacteria. BLAST searches of available fungal genome sequences with the sequences of these proteins suggest that homologous proteins are widespread in the fungal kingdom and occur in almost all fungal phyla including ascomycota, basidiomycota, zygomycota and glomeromycota [73]. It will be interesting to test whether these proteins are also involved in defense of these fungi against bacterial (or fungal) competitors and, if yes, to determine their specificity with regard to target organism. In this regard, it has recently been shown that few mutations can suffice to change the specificity of such a defensin-like protein [74]. On the other hand, members of the Csαβ-protein superfamily have been shown to exhibit a large variety of biological activities, including signaling functions, in invertebrates [75]. An endogenous role in signaling was also proposed for a fungal Csαβ-protein from *Aspergillus nidulans* [32].

### 3.2. Lysozymes

Plants and animals defend themselves against invading bacteria by producing enzymes hydrolyzing the β-1,4-glycosidic bond between GlcNAc and MurNAc in bacterial peptidoglycan [76,77]. These enzymes are generally referred to as lysozymes or muramidases and also occur in bacteria where they are needed for peptidoglycan reconstruction during cell growth. It should be mentioned though that the antibacterial activity of some of these enzymes has been shown to not solely depend on their enzyme function [78,79]. Animal lysozymes are classified in i(nvertebrate)-, c(hicken)- and g(oose)-types and some of the i-type lysozymes are probably rather involved in digestion of bacteria than antibacterial defense [80]. Plant lysozymes are structurally not related to the animal enzymes and always show also chitinase activity [81]. To our knowledge, thus far, only a single lysozyme of fungal origin, that gave rise to the so-called ch(alaropsis)-type of lysozymes, has been characterized in more detail [53]. This enzyme from *Chalaropsis sp.* belongs to the glycosylhydrolase family 25 (GH25) in the CAZY database (PF01183 in the PFAM database) along with homologous enzymes from other fungi, bacteria and bacteriophages. The secretome of *Agaricus bisporus*, which harbours four genes encoding putative GH25-type lysozymes in its genome, was shown to have lysozyme activity [82,83] but it is not clear whether the activity is due to these proteins. BLAST searches of fungal genomes with members of another family of bacterial and bacteriophage lysozymes (GH24/PF00959) suggest that fungi express representatives of at least two families of so-called microbe-type lysozymes. In contrast, the available fungal genomes do not appear to encode for homologs of animal or plant lysozymes. It remains to be shown whether the two families of fungal lysozyme-like proteins are involved in the defense against bacterial competitors.

### 3.3. Ceratoplatanins

The ceratoplatanin (CP) family is a group of secreted, small and cysteine-rich fungal proteins that has been implicated in fungus-host interactions [54,84]. The length of the mature proteins is 100–130 amino acids and shared features of the sequence are highly conserved N- and C-terminal signature sequences and four cysteine residues forming two disulfide bridges. The proteins were identified in the secretomes of plant pathogenic fungi and shown to exhibit phytotoxic activity and to elicit a defense response in the host plant [85]. Based on structural similarities to plant and bacterial expansins, fungal and animal endoglucanases and bacterial lytic transglycosidases, CPs may carry out a cell wall glycan-related function [86]. In agreement with the structural similarity to lytic transglycosidases targeting bacterial peptidoglycan, CPs were shown to exhibit, similar to LysM-proteins, binding activity to N-acetyl-glucosamine-containing oligosaccharides [55]. Interestingly, residues involved in this activity are well conserved in the protein family. In addition to this putative glycan-related function, several members of the CP family have been shown to be able to form self-aggregates *in vitro* at air/water interfaces similar to hydrophobins [85]. It remains to be tested whether these proteins play a role in the defense of fungi against fungal or bacterial competitors besides their function in the interaction with plants.

### 3.4. Lectins

There are only few reports about lectins affecting bacterial growth or viability by binding to bacterial surface glycans. Examples of characterized bacteriostatic or bactericidal lectins from animals bind either to glycoepitopes conserved in all bacteria, such as peptidoglycan or lipid A [87,88] or to highly variable glycoepitopes on the surfaces of Gram-negative and Gram-positive bacteria *i.e.*, the polysaccharide portion of LPS and LTA, respectively [89,90,91]. There is evidence for membrane permeabilization upon binding of these lectins to the bacterial surface [92,93], but the exact mechanism of lectin-mediated toxicity against bacteria is not clear. To our knowledge, however, no antibacterial lectins from filamentous fungi have been identified so far. Tectonin2 (Tec2) from the ectomycorrhizal mushroom *Laccaria bicolor*, a lectin with structural and functional homologs in horseshoe crabs, was recently shown to agglutinate *E. coli* strains carrying a specific glycoepitope on their O-antigen [20]. However, no Tec2-mediated growth inhibition of these bacteria was observed and hence it is not clear at the moment whether this recognition is physiologically relevant for the fungus.

## 4. Fungal Defense Effector Proteins Targeting Metazoan Glycoepitopes

In contrast to the glycoepitopes of fungal and bacterial competitors targeted by fungal defense proteins, the target glycoepitopes of metazoan predators are usually not directly exposed to the environment but located on epithelial surfaces in the lumen of their digestive tract (Figure 1). In addition, these epitopes are usually less conserved and not essential for the viability of the antagonist [18]. Despite these differences, binding of these glycoepitopes by fungal defense proteins has been shown to lead to severe impairment of several metazoan (and protozoan) predators ranging from inhibition of development to killing [56,62,94] (Table 1).

### 4.1. Hololectins

Most of the fungal defense proteins targeting metazoan glycoepitopes are carbohydrate-binding proteins devoid of other activities, commonly referred to as hololectins [17]. Many of these proteins were identified from dikaryotic fungi and are commonly referred to as fruiting body or mushroom lectins due to their abundance in the reproduction structures of these organisms [95,96,97,98]. These lectins lack a classical signal sequence for secretion and are synthesized on free ribosomes in the cytoplasm. Latter compartment is a perfect storage place for defense proteins destined to find their targets in the digestive tract of the predator upon ingestion [47]. Fruiting body lectins are small, highly soluble and stable proteins, belong to different lectin fold families and contain several binding sites for the same glycoepitope, either on the same or several subunits [99]. Characterized examples are AAL from *Aleuria aurantia* [47,48,49], XCL from *Xerocomus chrysenteron* [47,56,100] and its homolog TAP1 from *Sordaria macrospora* [47,57], CCL2 from *Coprinopsis cinerea* [19] and its structural homologs CNL from *Clitocybe nebularis* [47,58], MPL from *Macrolepiota procera* [59], SSA from *Sclerotinia sclerotiorum* [47,60] and RSA from *Rhizoctonia solani* [61,62,63], CGL1 and CGL2 from *Coprinopsis cinerea* [18,47] and Tectonin2 (Tec2) from *Laccaria bicolor* [20]. Depending on their carbohydrate-specificity, which can differ also between members of the same lectin fold family, the specificity of these lectins with regard to their target organisms can vary significantly [47]. Accordingly, the availability and the concentration of the targeted glycoepitopes in the intestinal epithelium were shown to correlate with the toxicity of the respective hololectin in nematodes [57]. In case of fruiting body lectins demonstrated to be toxic to the model nematode *Caenorhabditis elegans*, the glycoepitope targeted by these lectins *in vivo* could be identified using a genetic approach [18,19,57]. Most of these nematotoxic hololectins target epitopes on asparagine-bound glycans (N-glycans) of glycoproteins on the intestinal epithelium of the nematode (Figure 2). Interestingly, the targeted glycoepitopes are often specific for nematodes and some additional invertebrate phyla [19]. As an example, the Gal-β1,4-Fuc epitope present on some nematode N-glycan cores and targeted by the nematotoxic *C. cinerea* galectins CGL1 and CGL2, has also been identified in platyhelminthes [101]. Similarly, 2-O-Me-fucose and 3-O-Me-mannose residues on N-glycan antennae targeted by nematotoxic *L. bicolor* Tec2 as well as α1,3-fucosylated N-glycan cores targeted by nematotoxic *C. cinerea* CCL2, have been identified in molluscs and/or insects [102,103]. This broad specificity is advantageous for the fungus because it enables it to defend itself against a large number of predator species and phyla by a comparably low number of defense proteins. Interestingly, some of the glycoepitopes targeted by fungal hololectins are also targeted by endogenous hololectins of the metazoan predator [104] which may have implications for the toxicity mechanism of this class of fungal defense effector proteins (see paragraph 5).

### 4.2. Chimerolectins

In addition to above mentioned hololectins, several chimerolectins from fungi exhibiting toxicity towards metazoa have been characterized. These proteins consist of a lectin domain, usually of the RicinB(β-trefoil)-type, fused to a non-lectin domain [17]. The best characterized example is MOA from the fairy ring mushroom *Marasmius oreades* [66,105]. This protein consists of an N-terminal RicinB-type lectin domain fused to a cysteine-protease domain and forms a dimer in solution [65]. The protein is highly toxic to *C. elegans* and both the lectin domain and the catalytic domain are necessary for full toxicity [64]. Nematotoxicity of MOA was shown to be mediated by binding of the protein to Gal-α1,3-GalNAc epitopes on glycosphingolipids in the apical membrane of the *C. elegans* intestinal epithelium. Based on the pH- and Ca^2+^-requirements of the cysteine protease activity, it has been hypothesized that the protein is internalized and cleaves essential proteins in the lumen of the endoplasmic reticulum [64]. MOA homologs can be found in several other mushrooms. The homolog PSL from *Polyporus squamosus* has apparently lost its catalytic and nematotoxic activity but changed its specificity towards sialic acid-containing glycans [64,106]. Another well characterized fungal chimerolectin is LSL from *Laetiporus sulphureus* [67]. This protein consists of a N-terminal RicinB-domain and a C-terminal pore-forming domain and exhibits hemolytic and hemagglutinating activity. The lectin domain has an *in vitro* specificity for β-galactosides but the *in vivo* target is not clear [68]. Based on the structural similarity of the pore-forming domain to aerolysins, a bacterial β-pore forming domain, it is hypothesized that the protein oligomerizes to a hexameric pore complex upon binding to glycoproteins or glycolipids on a cellular membrane [107,108].

## 5. Mechanism of Toxicities Mediated by Glycan-Protein Interaction

Although not all of the glycoepitopes targeted by above mentioned fungal defense effector proteins (Table 1) have been identified unequivocally, at least two targeting strategies emerge: (1) The fungal defense effector proteins target highly conserved glycoepitopes of the antagonist exemplified by the intermediates of the biosynthetic pathways of cell wall polysaccharides in fungi or bacteria. Mere binding and thereby sequestering of these intermediates by the fungal defense proteins can interfere with the remodeling or biosynthesis of the cell wall and thus with the viability of the fungal or bacterial cells. It has recently been shown that blocking individual components of the bacterial cell wall biosynthesis machinery can cause futile cycles that are lethal for the cell [12]. As an alternative to targeting cell wall biosynthesis intermediates, some fungal effector proteins target highly conserved glycolipids e.g., fungal glucosylceramide or mannosylinositol-phosphorylceramide (MIPC), and thereby interfere with the function of these lipids in the cell membrane. As a possible toxicity mechanism, the effector proteins might sequester these glycosphingolipids from specialized domains in the fungal plasma membrane. Such domains have been shown to be important for the function of many integral membrane proteins [109]. (2) The fungal defense proteins target highly variable glycoepitopes of the antagonist e.g., LPS and LTA on the surfaces of Gram-negative and Gram-positive bacteria, respectively, or protein- or lipid-bound glycans on the surface of the intestinal epithelium of metazoans. The toxicity mechanisms of these interactions are unclear, however. In case of the toxicity of fungal hololectins binding to metazoan glycoepitopes, the multiplicity of binding sites for these glycoepitopes (multivalency) on the lectin appears to play a key role [110]. It has been hypothesized that the binding of endogenous multivalent lectins to glycoproteins carrying multiple glycans leads to the formation of glycoprotein-lectin-lattices on the cell surface [111,112]. These lattices are probably important for the function of the plasma membrane as they trigger or inhibit endocytosis and thereby determine the levels of the respective glycoproteins on the cell surface [113,114,115,116]. Accordingly, fungal multivalent defense lectins, that bind to the same glycoepitopes as the endogenous lectins involved in these lattices, may interfere with the formation of these lattices on the intestinal epithelial membranes of metazoan predators and thereby impair the function of these membranes e.g., with regard to nutrient uptake. Alternatively, crosslinking of glycosylated cell surface receptors by fungal defense lectins may elicit intracellular signaling of these receptors in the absence of a ligand [63]. Fungal chimerolectins probably use another mechanism since they bind, analogously to many bacterial toxins [117], to monovalent glycolipids. In this case, the binding to the glycolipid is thought to bring the ‘business’ (non-lectin) part of the protein in proximity of the cellular membrane from where the protein then either enters the cell to find a second, intracellular target, or alters the function of the cellular membrane directly e.g., by forming pores. 

## 6. Regulation of Fungal Defense Protein Biosynthesis

The biosynthesis of proteins involved in defense is usually tightly regulated because these proteins are not essential for the viability of an organism and their biosynthesis needs a lot of resources which are often limited [118]. Similar to plants, in the absence of an antagonist, the biosynthesis of many of above mentioned fungal defense effector proteins is developmentally regulated. Accordingly, a genome-wide gene expression analysis of the vegetative mycelium and young fruiting bodies of *C. cinerea* revealed that the antibacterial copsin is almost exclusively produced in the vegetative mycelium whereas most of the *C. cinerea* genes coding for insecticidal and nematicidal hololectins are specifically expressed in the fruiting body [119]. This spatiotemporal regulation results in a very efficient protection of specific fungal tissues against the physiologically most relevant antagonists, *i.e.*, vegetative mycelium against microbial competitors and fruiting bodies against metazoan predators, because the defense effectors are already in place when the antagonist attacks the fungus. On the other hand, at least some of the hololectin-encoding genes directed against metazoan predators were shown to be induced in the *C. cinerea* vegetative mycelium when this tissue was challenged with a fungivorous nematode [47]. Similarly, challenge of the vegetative mycelium of *Aspergillus nidulans* with actinobacteria and arthropods led to the induction of various gene clusters coding for the biosynthetic machineries of antimicrobial and cytotoxic secondary metabolites, respectively [120,121,122]. In case of the arthropod challenge, this treatment was shown to lead to induced protection of the fungal mycelium from grazing by the arthropod [121,122]. These results suggest that at least dikaryotic fungi possess, in addition to a constitutive, tissue-specific defense, also an inducible defense. In the course of this inducible defense, fungi appear to be able to recognize the type of antagonist they are confronted with and mount a corresponding defense response. The signals, receptors and downstream signaling pathways involved in this process are not known. Based on the few available reports, peptidoglycan fragments appear to be signals by which fungi are able to detect bacteria, possibly using intracellular receptors [123,124]. Similarly, ascarosides, pheromones produced by nematode to control their development, were reported to induce the formation of nematode traps in nematophagous fungi [125]. In other cases, physical contact between the fungus and the antagonist appeared to be necessary for induction [47,120]. Antagonist-dependent induction of defense genes in *A. nidulans* was shown to involve histone acetylation suggesting the involvement of epigenetic mechanisms [126].

## 7. Evolution of Fungal Defense Proteins

Studies on the toxicity of fruiting body lectins in the model nematode *C. elegans* have shown that a loss-of-function mutation in a single gene, e.g., coding for a glycosyltransferase involved in the biosynthesis of the targeted glycoepitope, can lead to complete resistance of the nematode to such a lectin [18]. Thus, it is relatively easy for the nematode to escape from a single nematotoxic lectin simply by altering its glycoepitopes in the intestine if these epitopes have no important endogenous function for the nematode. The fungus, on the other hand, can counteract this escape mechanism of the nematode by producing a cocktail of different lectins targeting different glycoepitopes of the same nematode. Accordingly, at least two characterized hololectins, CGL1/2 and CCL1/2, with nematotoxic activity and different carbohydrate-specificity are known to be expressed at the same time in the fruiting body of *C. cinerea* [119]. Fungi have different possibilities to diversify the spectrum of defense effector proteins. One possibility is the multiplication of existing defense genes which allows altering the function of individual gene copies by spontaneous mutations. An example for this mechanism is probably the tandem duplication of the CGL1 and CGL2 genes in the genome of *C. cinerea* [127]. This duplication must have happened very recently because the gene sequences including the non-coding regions are highly homologous and the carbohydrate-specificity of the two encoded hololectins is identical. In addition to these two gene copies, the *C. cinerea* genome harbors a third gene copy coding for a hololectin, CGL3 that shows a lower degree of similarity and has an altered carbohydrate-specificity [128]. So far, however, no toxicity of this hololectin could be demonstrated [47]. Another possibility to acquire additional defense genes is horizontal gene transfer (HGT). Several cases of HGT in fungi have been described [129], including the horizontal transfer of entire gene clusters for toxic secondary metabolites between fungi [130]. Besides other fungi, bacteria appear to be the main source of fungal genes acquired by HGT [129,131,132]. To date, only one gene coding for a fungal defense effector protein, the chimerolectin LSL from *L. sulphureus* and its homologs in *C. cinerea*, has been reported to be acquired by HGT from bacteria [133]. The lack of introns in the coding regions of many fungal defense genes, the occurrence of homologous genes in bacteria and the patchy distribution of the genes within the fungal kingdom may be indications for acquisition of these genes by HGT from bacteria.

## 8. Conclusions and Outlook

Cell surface glycoepitopes are a universal feature of all living cells [134]. They are easily accessible, structurally very diverse and conserved between cells/organisms of the same taxon or sometimes of different taxa. Thus, glycoepitopes represent a kind of molecular barcode on the surface of every cell/organism and are therefore a preferred target of defense effector proteins to combat antagonists of a specific taxon or of several taxa. The evolution of proteins targeting glycoepitopes that are conserved between organisms of the same taxon or of different taxa, increases the spectrum of antagonists that can be addressed with the often limited number of innate defense effector proteins of an organism. The variability of a specific glycoepitope inversely correlates with the essentiality of its function for the viability of the host organism. In this regard, glycoepitopes of structural components of fungal or bacterial cell walls are usually less variable than glycoepitopes of glycoproteins and glycolipids on the surface of metazoan cells. Invariable glycoepitopes, e.g., peptidoglycan of bacterial cell walls, are also highly conserved between different organisms of a specific taxon, e.g., bacteria. Such invariable and highly conserved glycoepitopes are exquisite targets of fungal defense effector proteins since (1) mere binding of the effector to such a glycoepitope is likely to interfere with an essential process, and (2) all organisms displaying this glycoepitope on their cell surface can be targeted by the same effector protein. Microorganisms counteract this potential Achilles heel by masking their conserved surface glycoepitopes with highly variable glycoepitopes, e.g., LPS and LTA in case of Gram-negative and Gram-positive bacteria, respectively. Interestingly, no defense effector proteins of fungi targeting these highly variable glycoepitopes have been identified so far. In case of metazoan cells, invariable glycoepitopes are rare. Most of the metazoan glycoepitopes targeted by fungal defense effector proteins, e.g., N-glycans on the intestinal epithelia of nematodes, are variable but often conserved within a specific taxon, e.g., nematodes, and sometimes beyond, e.g., insects or molluscs. Since these glycoepitopes do not appear to be essential for the viability of the cell/organism, it is surprising that mere binding by fungal effector proteins (hololectins) can lead to cytotoxicity. The exact mechanism of this hololectin-mediated cytotoxicity is unclear and remains one of the main open questions in glycobiology. Answering this question in case of one of the fungal hololectins would require a genetically tractable invertebrate cell line [63]. The toxicity mechanism is more evident in case of the fungal chimerolectins where binding of the defense effector proteins to glycoepitopes on the cell surface does not result in toxicity directly but brings the protein close to the plasma membrane from where the protein is inserted into the membrane or endocytosed to meet intracellular targets. It will be interesting to see whether the binding of fungal chimerolectins to glycosphingolipids always correlates with endocytosis and intracellular trafficking of these proteins as has been suggested for bacterial toxins [117].

Fungal defense effector proteins have to bind their target glycoconjugates under harsh conditions. In case of the secreted effector proteins directed against fungal and bacterial competitors, these proteins have to withstand extreme conditions with regard to pH, temperature, salt, humidity and microbial proteases. For this reason, a common feature of many of these proteins is the stabilization of their fold by several disulfide bridges. The recently characterized antibacterial Csαβ-defensin copsin from *C. cinerea*, whose mature form consists of 57 amino acids, is stabilized by six disulfide bridges [52]. Fungal defense effector proteins directed against metazoan predators, on the other hand, have to resist digestive proteases in the intestine of the predators. These proteins cannot be stabilized by disulfide bridges since they are synthesized under the reducing conditions of the cytoplasm. Probably for this reason, these proteins are often synthesized along with protease inhibitors in the cytoplasm of these fungi [135]. Since some of these protease inhibitors, e.g., Cospin from *C. cinerea* [136], have been shown to exhibit toxicity towards some of the same organisms as the fungal defense lectins, they may have the dual function of protecting the accompanying fungal defense lectins from proteolysis by intestinal proteases of the predator and exerting an antinutritional effect adding up to the lectin-mediated toxicity. According to a recent report, the two types of fungal defense proteins may even be able to form complexes [137]. The physiological significance of such complexes has still to be demonstrated, however.

The current knowledge about glycan-binding proteins as effectors of the innate defense system of multicellular fungi against antagonists opens interesting avenues for future research on this topic. Based on the differential expression of some genes coding for fungal defense effector proteins upon challenge with bacteria and nematodes, it will be possible, on the one hand, to identify novel, antagonist-specific candidate defense effector proteins of these organisms. The molecular characterization of these effector proteins may reveal novel antimicrobial and cytotoxic mechanisms and targets, which could serve as leads for the development novel antibiotics, antifungals or antihelminthics to be used in clinics or crop protection. On the other hand, further analysis of the signals, receptors and signaling pathways responsible for this differential gene expression will give insight into the molecular mechanism of the crosskingdom communication between fungi and bacteria or nematodes and, by comparison with analogous mechanisms in plants and animals, in the evolution of innate defense systems in multicellular eukaryotes in general.
